# Medical Students’ Predictions as to How Medical Practice Will Evolve by the Year 2050

**DOI:** 10.7759/cureus.98347

**Published:** 2025-12-02

**Authors:** Sheema Chaudhry

**Affiliations:** 1 Internal Medicine, Princess Alexandra Hospital, London, GBR

**Keywords:** artificial intelligence, focus group interviews, fourth-year medical students, future career, medical education curriculum

## Abstract

Background

Medicine is a field that is continually evolving, and sociopolitical events, medical advances, and challenges have contributed to the development of medicine over time and will continue to impact the future. There is current literature relating to the future of medicine focusing on technology, medical education, and the changing role of doctors.

Aims

This study aims to explore medical students’ perceptions of how medical practice may be in the year 2050 and to utilize this information to guide potential changes that could be made to the medical school curriculum in order to best prepare students for these changes.

Methods

General qualitative methodology with a constructivist and interpretivist standpoint was used to conduct semi-structured focus groups using medical students at the University of Nottingham. The data were transcribed and analyzed using thematic analysis.

Results

Seven key themes were derived from the data: Robotics and Artificial Intelligence (AI), Technology, Genomics, The Future of the National Health Service (NHS), Epidemiological Uncertainty, The Changing Role of a Doctor, and Thoughts on How to Prepare for the Future.

Conclusion

The seven themes derived from the data represent the students’ thoughts on how medical practice will change by the year 2050 when they are practicing. This can be organized again into sociopolitical events, medical advances, and challenges to provide a broad image of how medicine may be and thus how medical students can be prepared in medical school for their careers.

## Introduction

This study explores how medical students perceive the practice of medicine by the year 2050 through the study of the evolution of medicine from historical foundations to the future possibilities. Medicine has been forged by societal, economic, and technological forces. In the early days of modern medicine, healthcare was delivered through a diverse array of practitioners, including barber-surgeons, midwives, and apothecaries [1]. Care was unregulated, leading to inconsistent quality and outcomes, which prompted the establishment of the Royal College of Physicians in 1518. This marked the beginning of professional regulation, transforming the role of the physician and establishing standards of care [2].

The 20th century brought several monumental events, changing the face of medicine, such as the World Wars that led to advances in trauma management, infection control, and surgical techniques [3]. Following the war, as population size increased rapidly, social reforms such as the acceptance of women into the Royal College of Physicians demonstrated the transformation and progression of the delivery of healthcare in the UK [4]. The establishment of the National Health Service (NHS) in 1948 provided universal access to healthcare, reducing inequalities and ensuring that medical advances benefited the broader population [5]. There were also several innovations, including the discovery of penicillin and anesthesia, which allowed for previously fatal conditions to be treated, thus expanding the possibilities of medical and surgical intervention [6]. These historical developments demonstrate how medical practice has evolved in response to societal needs and challenges over a vast timeframe.

Currently, healthcare systems such as the NHS are facing significant pressures, such as funding constraints, workforce shortage, and the challenges associated with the ageing population in the UK [7]. On a global scale, public health challenges such as antibiotic resistance and the rise of chronic diseases, including obesity and diabetes, demonstrate growing issues faced by the international medical community and the need for adaptive strategies in response to immediate and long-term demands [8].

The practice of medicine in the future is poised for potential monumental changes, driven by artificial intelligence (AI), advances in technology, and genomics [9]. Robotics and AI are projected to improve diagnostic accuracy and speed, streamline surgical procedures, and even reshape clinical roles and the way healthcare is delivered in the future [9]. The use of telemedicine, an area that has already been explored during the COVID-19 pandemic, will continue to redefine access to healthcare, despite the challenges posed in data security, confidentiality, and professional training [9].

Future healthcare is also projected to face further societal and environmental challenges [10]. Climate change has been identified as a key factor in impacting both physical and mental health through extreme weather, shifts in infectious disease patterns, and air quality [10]. Furthermore, advances in biotechnology bring both opportunities and risks, including the potential misuse of gene editing and the emergence of novel pathogens [10].

Understanding the trajectory of modern medicine, through exploring historical milestones, current issues faced, and the projected innovations and concerns, provides insight into the ever-evolving role of healthcare professionals and future demands of medicine. By examining these trends and exploring the thoughts of medical students, this study aims to illuminate the expectations, challenges, and opportunities that will define the next era of medicine.

Research aim

This study aims to investigate how medical students perceive the practice of medicine in 2050 and how medical education can best prepare students for future healthcare challenges.

Research objectives

This study aims to (1) examine medical students’ perceptions of future medical practice in specific domains, including technological advancements, workforce roles, models of care, evolving disease patterns, ethical considerations, and patient-centered approaches; (2) explore students’ views on the implications of these changes for medical education, including curriculum design and preparation for future healthcare roles; (3) identify factors perceived by students as likely to influence the evolution of medical practice, including sociopolitical trends, global health challenges, technological innovations, and changes in healthcare delivery; and (4) generate insights that can inform the alignment of medical curricula with anticipated future needs, ensuring that graduates are equipped with the knowledge and skills required for 2050 and beyond.

Research question

This study examines the following research question: How do medical students think medical practice will change by 2050?

## Materials and methods

This study adopts a qualitative approach grounded in interpretivist and constructivist philosophies, focusing on participants’ subjective experiences and perceptions of how medicine may evolve by 2050. The study’s philosophical foundation reflects the belief that knowledge is shaped by individual experience and interpretation. Interpretivism emphasizes understanding multiple subjective realities, while constructivism highlights how individuals form meaning through reflection and interaction [11]. Together, these frameworks support a rich exploration of medical students’ expectations for the future of healthcare and medical education [11].

Focus groups were chosen as the primary data collection method to encourage collaborative discussion, allowing participants to exchange ideas, challenge perspectives, and build on each other’s responses [12]. There were two focus groups, each comprising six participants, as the optimal size for focus groups is between 5 and 8 to promote discussion between the individuals [12].

The focus groups were conducted at the University of Nottingham in November 2019. The medical school hosts a five-year undergraduate medical program and a four-year graduate entry route. Purposeful sampling was used, and all medical students (N=2,577) were invited to participate via poster advertisements around the University of Nottingham Medical School campus and an email sent to all students of the medical school. Participants were included if they were currently enrolled medical students in years 3-6 and were willing to discuss future medical practice. Students were excluded if they had previously participated in a formal study on medical futures or were not fluent in the language of discussion. The first 12 participants who signed up were selected to participate as they were all deemed to be suitable participants by the research team. The participants were female third-year medical students, 10 from the undergraduate course and two from the graduate entry course. The focus groups were conducted in the medical school library.

Ethical approval was received from the University of Nottingham’s School of Medicine Research and Ethics Committee (reference number: 378-1908). Prior to participation, the purpose of the focus groups was explained in detail, and participants were given opportunities to ask questions before signing consent forms. The focus groups were audio-recorded using digital voice recorders. Transcription was done using human transcription within 24 hours, after which the recordings would be permanently deleted. To maintain confidentiality within the group setting, all participants were reminded of their responsibility to respect the privacy of others and to refrain from sharing any information discussed outside the session.

Measures were also taken to manage potential power dynamics, including the researcher adopting a neutral facilitation style and emphasizing that all contributions were voluntary, with no impact on academic standing or relationships within the cohort. Participants were told that they could request access to their own transcripts at any time, although no participants requested this information, and it was made explicit that they would have the opportunity to review and clarify their contributions if they wished. All names and identifiable details were anonymized during transcription to further ensure confidentiality.

Focus group sessions

Two focus group sessions were conducted, lasting 40-60 minutes each, following the guidelines as described by Krueger and Casey [12]. The researcher was the facilitator for the focus groups. A semi-structured discussion guide with pre-determined probes and follow-up questions was used to ensure consistency across groups. The facilitator encouraged equitable participation, managed dominant speakers, and ensured quieter participants were invited to contribute. The purpose of the focus groups was explained to all participants beforehand, with opportunities to ask questions, and consent was obtained to voice-record the sessions. The aim of the focus groups was to explore as many different ideas from the students and be able to build upon the answers to come up with thoughts on how they expected medicine to be by the year 2050 and how they can be best prepared to work as doctors in this time.

In order to generate the questions, the researcher conducted some prior research in the area to gain knowledge on what areas the participants may discuss. The review by Topol highlights some of the key advances in medicine, and so, particular interest was shown in the areas mentioned in the report [13]. Some general questions were chosen in order to gauge the initial ideas as to what participants thought medical practice would be like in 2050.

Specific follow-up questions were asked utilizing the knowledge from the research on the areas participants discussed. Some questions relating to the current curriculum were asked in order to gauge how participants thought the curriculum could be adapted to best prepare medical students for their future careers. The moderator asked the questions and clarified any ambiguities to the participants.

After two sessions, the sample provided sufficient information to address the aims of this study, and thus, no further focus groups were arranged. This was evidenced by the fact that the second focus group yielded no major new themes, only minor variants or elaborations. Thus, there was a new-idea plateau. The questions used in the focus groups can be found in the Appendices. These questions were chosen using the research from existing knowledge, and general questions were chosen to gain an overall perspective of how the students perceive medical school. The questions were standardized and asked to both groups in the same order as outlined in the Appendices.

The focus group transcripts were analyzed using thematic analysis. Thematic analysis is the process of identifying patterns and themes within qualitative data [14]. Thematic analysis is a very flexible method because it is not tied to a particular epistemological perspective [14].

The data were manually coded by a single researcher following Braun and Clarke’s six-phase framework [15] using an inductive approach. During the theme refinement phase, initial codes were iteratively reviewed, compared, and clustered to ensure that emerging themes accurately reflected the data rather than the coder’s assumptions. Possible identified themes were checked for internal coherence and distinctiveness, and extracts were revisited to confirm that theme boundaries were conceptually sound. Although the analysis was undertaken by one coder, methodological rigor was strengthened through informal peer debriefing, in which emerging themes and coding decisions were discussed with a qualitative research-experienced colleague to challenge interpretations and minimize bias. However, formal triangulation or multi-coder analysis was not conducted, which represents a limitation of the study.

In this research, all of the participants were female third-year medical students; demographics other than sex and year of study were not collected. There were two focus groups containing six students each. This may have had an influence on the data collected, as having the participants from the same cohort and sex may mean that they have similar experiences within medical school, resulting in unrepresentative results. The researcher also came from the same cohort and knew two of the participants before the focus groups were conducted. This could further compromise the results, as it may have led the participants to only discuss information that they thought the researcher would understand or find interesting or beneficial. The researcher, being from the same background as the participants, would however mean that there was an equal balance of power, as the participants would be more likely to feel at ease as they could relate to the researcher.

## Results

Initial findings

The data was initially familiarized with prior to generating the initial code as per Braun and Clarke’s six-phase framework [15]. Table 1 shows the initial findings generated from the data.

**Table 1:** Initial Findings NHS: National Health Service, AI: artificial intelligence

Initial findings
The NHS
Privatization
Technology
Strain on the NHS
Robotics
Genomics
Increased efficiency due to technology
Personalized medicine
Personalized medicine improving treatment
Personalized medicine improving diagnosis
Mobile applications
Online consultations
Antibiotic resistance
Politics
Digital health interventions
Importance of the clinical phase
Relevance of epidemiology
Relevance of public health
The level of detail of the curriculum
How psychology is taught
Focus on the patient
Costs
Vaccines
Patient-centered care
Mental health being different now
Artificial intelligence
Doctors having common sense that AI might not
Wearable technology increasing efficiency for doctors

Following this, initial themes were searched for as per phase 3 of Braun and Clarke’s framework (Table 2) [15]. 

**Table 2:** Initial Themes NHS: National Health Service, AI: artificial intelligence, HIV: human immunodeficiency virus

Ideas	Initial theme
The NHS, privatization, politics, strain on the NHS, costs	The NHS currently
Increase technology, new areas being interesting, genomics being difficult to learn, the relevance of learning some areas early on	Thoughts on the future
Technology reducing strain on the NHS, privatized NHS	The NHS in the future
Robotics doctors having common sense that robotics might not, surgery robots	Robotics
Artificial intelligence, AI not being used currently, AI being able to diagnose, not having exposure to AI, doctors having common sense that AI might not	Artificial intelligence
Technology, online consultations, digital health interventions, mobile applications, online consultations, digital health interventions	Types of technology
Understanding technology, online consultations increasing efficiency, wearable technology increasing efficiency for doctors, expense of wearable technology, technology can save lives, efficiency of medicine being increased by technology, online consultations increasing efficiency, age impacting the use of technology, wearable technology increasing efficiency for doctors	Effects of technology
Genomics, personalized medicine, religious objections to personalized medicine, expense of genetic testing, learnt a small amount of genomics already, personalized medicine, improving treatment, personalized medicine, improving diagnosis, genomics is a difficult subject	Genomics
Obesity increasing, diabetes increasing, HIV increasing, aging population diseases increasing, dementia increasing	Increasing issues
Cancer, obesity-related diseases, age-related diseases, musculoskeletal-related diseases, dementia, diabetes, HIV	Predictions for what may be more prevalent
Empathy being provided by humans, humans having qualities that machines do not	Human characteristics that machines do not have
Cancer being less prevalent, vaccines curing diseases, improvements in cancer care	Predictions for what may be less prevalent
Importance of the clinical phase, relevance of epidemiology, relevance of public health, how psychology is taught, the level of detail of the curriculum, relevance of learning technology, genomics and artificial intelligence early may not be useful	Thoughts on the current curriculum
Dealing with antibiotic resistance, dealing with antibiotic resistance	Antibiotic resistance being an issue
Patient-centered care, mental health being different now, focus on the patient	How medicine is delivered differently, positive outcomes
Preparing for the future being important, new areas being interesting	Thoughts on/preparing for the future

As part of phase 4 of Braun and Clarke’s framework, the themes were then reviewed [15]. Table 3 shows the initial themes that were derived from Table 2.

**Table 3:** Initial Themes Numbered NHS: National Health Service

Theme number	What it contains
1	NHS currently and NHS in the future
2	Robotics and artificial intelligence
3	Types of technology and effects of technology
4	Genomics
5	Increasing issues, predictions for what may be more prevalent, and predictions for what may be less prevalent
6	How medicine will change and how medicine has already changed
7	Thoughts on the current curriculum and thoughts on the future

Final themes

Table 4 demonstrates the final themes that have been defined and named from the thematic analysis as phase five of Braun and Clarke’s framework [15]. The themes were as follows: Robotics and Artificial Intelligence, Technology, Genomics, The Future of the NHS, Epidemiological Uncertainty, The Changing Role of a Doctor, and Thoughts on How to Prepare for the Future. The themes are outlined further in Table 4.

**Table 4:** Final Themes NHS: National Health Service

Theme number	Theme title
1	Robotics and Artificial Intelligence
2	Technology
3	Genomics
4	The Future of the NHS
5	Epidemiological Uncertainty
6	The Changing Role of a Doctor
7	Thoughts on How to Prepare for the Future

Theme 1: Robotics and Artificial Intelligence

The students were aware of some of the current uses of robotics within surgery, and there was agreement that there would be a continual increase in the use of robotic surgery, potentially to the extent of replacing the current role of surgeons.

Student 1: “I think we will see more robotic technology being used, especially in surgery.” 

AI was an area linked to robotics that the students also believed would be something that would have an increased role, particularly within the role of diagnosis. Some of the participants gave specific areas in which they thought there would be an increased use of AI, including some specialties and within primary care.

Student 7: “I feel like artificial intelligence is going to take over big time.” 

The participants also considered some of the disadvantages, such as the inability of these devices to show emotions and thus form a relationship as humans can.

Furthermore, there were also doubts relating to the ability of the machinery to be able to correct mistakes. Due to this, it was agreed that technology and AI would be unable to replace the role of doctors, but would change some of the tasks that doctors are required to do.

Student 1: “They can’t ever replace the more emotional and personal side of being a doctor.”

Theme 2: Technology

Technology was an area in which the students had already experienced changes in medicine in their lifetime. It was a key factor in how all of the students thought medical practice would evolve in the future. There were several different areas of technology that were considered; however, some of the key areas of discussion were telemedicine and wearable technology.

Student 1: “I think technology is something that will really change this field.” 

Telemedicine was an area that some of the participants were aware of as something that was being implemented currently.

Student 2: “It will become more popular to do the online or app consultation because I know they’re introducing that in London.”

The participants were asked about their opinions relating to telemedicine, linked to the example of Babylon, which is an online general practitioner (GP) service. They were able to appreciate some of the advantages of using services like these.

Student 2: “It saves time, so doctors can like treat in person.” 

They were also able to consider potential drawbacks of these services, such as the lack of personalization and difficulties diagnosing virtually, thus potentially causing errors and being more inefficient than traditional GP services.

Student 6: “They don’t always have access to all medical records, so its possible that contraindications can happen.”

Using this information, the students were able to conclude overall that they did think that telemedicine services would increase in the future.

Student 5: “Like it would save a lot of money, costs, so I think it could be probably increased.”

Wearable technology was a key area that the Topol report predicted would play a large role in the future of medicine. Overall, the participants had varying ideas on how they thought wearable technology might be used.

Student 9: “I feel like a lot more people have Fitbits and Apple watches these days, and it links back to the whole technology advancing.”

The participants discussed some of the positive effects of wearable technology and mainly related them to increasing efficiency in a healthcare setting. Some of the other advantages included the interactive nature of wearable technology and also the potential to improve patients’ standard of living.

Student 4: “Yeah, you could monitor yourself and like if you see that your heart rate is high, like, maybe I need to go see someone.”

The only disadvantage that the participants associated with wearable technology was the high cost of the devices.

Student 4: “Yeah, like at the moment, the seizure alarm is so expensive, so you have to be in a certain position to afford it.”

Theme 3: Genomics

Genomics was an area in which the students already had some prior knowledge in as they had learnt about it in earlier years of medical school. It was a key area in which they predicted medicine would advance.

Student 1: “Genes and genomics is really interesting at the minute, too. Like, they’re talking of personalized genomics, which will completely revolutionize the field.”

Personalized medicine was the key area that the participants discussed; they also had opinions on some of the advantages of this.

Student 5: “Making treatments more personalized. I think, if you can figure out what genes may predispose somebody to something, you could help treat them from an earlier age.” 

The participants also reflected on some of the possible implications of using genomics in medicine, particularly relating to privacy concerns and religious objections that patients may have.

Student 6: “I think that there is a side of society that will never agree […] There is a real religious antagonist to this.”

Theme 4: The Future of the NHS

An important factor considered by the participants with regard to their future in practicing medicine was the status of the NHS. At the time the focus groups were conducted, it was before the general election, and thus, there were political uncertainties and queries about how the NHS may be run in the future.

Student 1: “I think privatization will definitely increase, and the handing out of NHS services to private companies.”

Funding was a key worry with regard to the NHS and how it would operate in the future, especially with being able to afford new equipment and services.

Student 3: “There have obviously been promises of putting more money into the NHS and building more hospitals and that kind of thing, so that would have an effect.”

Theme 5: Epidemiological Uncertainty

As medicine continually evolves and progresses, it is expected that the diseases that face the human population by 2050 may differ from what is currently present. The participants were initially asked what conditions they thought would be more prevalent in the future. Through looking at current trends in medicine, there was an overall consensus that diseases relating to the aging population and obesity would increase in prevalence in the future.

Student 1: “Diabetes and obesity, just because of our diets and lifestyle.” 

Another area that was discussed by the participants was the increase of antibiotic resistance in the future and how this could cause the return of some diseases that we are currently able to treat.

Student 4: “Antibiotic resistance as well, because people keep talking about it by like 2030 or something, antibiotic resistance is going to be across all areas.”

The participants were also asked about diseases that may have a reduced prevalence in the future. They were able to draw upon their current knowledge of what is being researched to estimate that diseases that have a vaccine will decrease in prevalence, as well as other diseases that are currently undergoing a lot of research, such as cancer.

Student 4: “It’s the stuff that they’ve got vaccinations for really so, like MMR, so that’s already starting to decrease because there’s a vaccine, but there’s an issue with.”

Despite these opinions, the participants were uncertain as to what diseases may be present by the year 2050.

Student 8: “I think it’s hard to predict because each decade, a new illness comes about.”

Theme 6: The Changing Role of a Doctor

As medicine has adapted and evolved over time, the role of doctors and what they are expected to do has also changed. The participants discussed how over time there has been a more patient-centered approach in medicine.

Student 3: “I think there’s been more of a focus on patient centered care.”

One of the participants discussed how other healthcare professionals’ roles had changed as they received more responsibilities, thus taking these jobs away from doctors and changing their roles.

Student 4: “Increasing the responsibilities of like nurses, I think practitioners like giving nurses the ones that aspire to go further then say pharmacists as well, giving them more of an opportunity.”

The participants expected the role of doctors to change further in the future. Through the use of robotics and AI, some of the participants believe that some roles of doctors currently, such as diagnosing and performing surgeries, may be taken over, thus increasing efficiency for doctors and allowing them to perform other tasks.

Student 12: “Things like diagnosis in like radiology or histology or something it could replace the role of those specialist.”

Theme 7: Thoughts on How to Prepare for the Future

The participants were asked questions relating to the current curriculum and whether they thought it was helping them prepare for their careers as doctors in the future. The importance of clinical practice was something that was highlighted by the students as they believed this was a crucial area in their medical education, and was perceived to be where they learn more skills relating to working as a doctor. The students expressed how their education could be improved by having more clinical exposure earlier in medical school.

Student 1: “I wish we got more opportunities to develop clinical skills in the pre-clinical phase.”

The participants were also asked if they found areas of the curriculum to be less helpful; there was a shared opinion that the depth of knowledge learnt in epidemiology and pathology was not necessary at such an early stage in their education.

Student 1: “I think epidemiology and public health […].” 

The participants were also asked if they thought that being taught the areas that they perceived to have a change in the way medicine is practiced in the future would be helpful. There were mixed opinions relating to this. Some of the participants thought it would be useful; however, some questioned whether medical school was the right time to learn about these things and how learning them later on could be more beneficial.

Student 5: “Yeah, I think if it’s going to be the future of medicine, I think we need to know more about it, so that we are prepared as much as we can.”

## Discussion

The data from the two focus groups have been transcribed and organized into seven key themes using thematic analysis. The results will be discussed, dividing them into socioeconomic events, medical advances, and challenges, as this will be able to provide an overall picture of how medicine is likely to be practiced in the year 2050. Importantly, the findings carry clear implications for medical education, given the study’s stated aim to inform potential curricular development.

Socioeconomic events

The status of the NHS with respect to privatization was a key factor that the participants believed would greatly impact careers. The possibility of privatization was seen as something that would “definitely increase” as they believed that there was already “handing out of NHS services to private companies.” As the focus groups were conducted before the 2019 General Election, there were increased worries due to the uncertainty that surrounded the NHS. Political events such as elections and Brexit may majorly impact healthcare in the UK. As mentioned in the introduction, the majority of drugs supplied to the UK come from or pass through the EU, so Brexit may mean that the sources of medical supplies change [16]. The participants agreed that the NHS requires an increase in funding to remain sustainable. The uncertainty surrounding the future of the NHS is also mirrored in literature, as there are many ideas that people have about how the NHS may develop, but there is agreement that the NHS is unsustainable if there are no changes in how it is supported currently. The long-term sustainability of the NHS and adult care report published by the House of Lords [17] highlights the necessity of increased funding in the NHS, either through higher taxation or a change in government allocation of funding. Some of the possible curricular implications include teaching on political influences on healthcare, preparing students to navigate system-level uncertainty, and health system education; such additions would better equip graduates for clinical decision-making within a shifting and increasingly complex healthcare environment.

Medical advances

Technology was identified by all participants as a key area shaping the future of medicine. The students were able to recognize the influence it has already had on medicine and predicted further progress: “We will see technology way more than we have ever seen it.” This aligns with literature highlighting rapid growth in artificial intelligence (AI), genomics, and digital health technologies such as telemedicine [9]. Telemedicine was an area that was discussed in depth, with participants anticipating its further use given the convenience and efficiency it provides: “It will become more popular to do the online or app consultation.” The benefits of telemedicine were also discussed, including improved accessibility, time savings, and reduced strain on NHS services, which is consistent with findings that telemedicine lowers non-attendance rates [18].

There were, however, concerns raised regarding weakened doctor-patient relationships and reduced empathy: “The lack of things like empathy … could make them not want to go to the GP in the future.” Participants also noted the potential for miscommunication and incomplete assessments [18]. Suggested solutions included expanding video consultations to preserve visual interaction. Despite some participants questioning the motives behind private telemedicine services, most agreed that the NHS’s increasing adoption could mitigate such issues. Overall, students viewed telemedicine as beneficial but requiring careful balance between efficiency and patient connection.

Participants envisioned AI becoming central to diagnosis and surgery, referencing current technologies such as “that robotic surgery arm in Derby.” They believed such innovations would expand due to their precision and efficiency, reducing trauma and recovery time [13]. Yet, concerns were expressed over robots’ lack of human judgment: “You need the aspect of common sense … are they trained in all the things that could go wrong?” Students recognized that while robots can enhance precision, human oversight remains essential [9].

In diagnostic fields, students suggested AI could play a major role in radiology and pathology, “Maybe … it could replace the role of those specialists or help them to come up with a diagnosis.” Studies support this, showing that AI can reach up to 96% diagnostic accuracy in radiology, comparable to human performance [19]. Participants viewed AI as a tool to enhance, not replace, doctors, freeing clinicians to focus more on patient care. The Topol report similarly highlights AI’s potential to automate administrative processes, improving system efficiency and patient management [13]. However, students demonstrated limited awareness of how these systemic changes might be implemented, underscoring a need for AI-related education within medical curricula.

Genomics and personalized medicine were areas that were recognized as growing fields in medicine, with all participants in agreement that genomics would become integral to prevention and treatment: “If you can figure out what genes may predispose somebody to something, you could help treat them from an earlier age.” The NHS is already establishing Genomic Medicine Centres to enable such approaches [19]. Students associated personalized medicine with earlier detection, targeted therapies, and optimized drug dosing, benefits which are supported by literature highlighting its proactive potential [20].

Despite this, ethical and social concerns were also noted. Some participants anticipated resistance to genomic testing: “There might be some sort of resistance … because it’s a very, very personal thing.” Others referenced religious objections: “There is a side of society that will never agree to the idea of genetic engineering.” These views echo existing debates on the moral implications of genetic testing [21]. Such diversity of opinion highlights the importance of maintaining a strong doctor-patient relationship to navigate sensitive ethical issues.

Overall, participants believed technology would transform medicine across diagnostics, treatment, and communication, yet they emphasized that human judgment, empathy, and ethical awareness would remain vital. As one participant summarized, technology will not replace doctors but “change the role of doctors,” allowing them to focus more on patient care while harnessing innovation responsibly.

The findings emphasize the importance of equipping future doctors with AI and digital literacy and training in remote consultation skills, especially building rapport and clinical judgement via telemedicine. These competencies are increasingly recognized by reports such as the Topol review [13], underscoring their relevance for curriculum committees and accreditation bodies. In addition to this, the above findings also demonstrate that strengthening genomic medical education, including the ethical implications and communication within the area, would support preparedness for a healthcare environment increasingly shaped by personalized interventions.

Challenges

Antibiotic resistance was identified by the participants as one of the greatest challenges facing future healthcare, with one describing it as “one of the biggest issues we’re going to face.” They were aware of its growing global concern, “people keep talking about it,” and also questioned whether new antibiotics could be developed quickly enough to combat resistance. The World Health Organization warns that by 2050, drug-resistant infections could cause up to 10 million deaths annually and push 24 million people into extreme poverty [20]. Current literature highlights the need for prudent antibiotic use and preventive strategies such as vaccines, genetically guided dosing, and novel drug development [22].

Epidemiological uncertainty was also raised, particularly in relation to the aging population within the UK. Students predicted increased prevalence of age-related diseases such as dementia, fractures, and myocardial infarction, consistent with projections that by 2050, conditions such as hypertension and arthrosis will rise sharply [23]. They expressed concern about how healthcare systems would cope, particularly given workforce shortages, which are estimated to reach 250,000 by 2030 [24].

Another major concern identified by the candidates was the growing obesity epidemic, attributed to be due to “our diets and lifestyle.” It is predicted that by 2030, 34% of men and 40% of women in the UK will be obese [25]. Participants linked this trend to rising rates of obesity-related diseases, including type II diabetes, coronary heart disease, and certain cancers. Evidence supports this, forecasting that such conditions could at least double in prevalence by 2050 [26]. These increases are expected to strain NHS resources, reduce workforce productivity, and raise welfare costs, potentially impacting both the healthcare system and wider economy [18].

Some of the curricular implications derived from these findings are enhancing training on public health, preventive medicine, and population-level trends, in addition to integrating antimicrobial stewardship education more explicitly across clinical years. These additions to medical school education would support the development of future doctors capable of addressing large-scale health threats.

Strengths and limitations

The strengths of this study include the use of focus groups to gauge the students’ opinions. The choice of medical students as participants is advantageous as it is highly relevant to curriculum reform and professional identity formation. The focus groups had no more than six participants, which meant that each person was given the time to express and discuss their opinions. By using medical students, it provided a first-hand experience of current opinions on the medical school curriculum and also provided an insight into how future doctors feel about what their careers may be like.

The main limitation of the study was the diversity of the participants. All of the participants were female third-year students from the same institution, meaning that the students were only able to speak from their limited experience in a clinical setting; therefore, their views may not have been representative of the entire medical school on topics such as education. Furthermore, as participants were all from the same institution, their perspectives may reflect local institutional culture. In addition to this, due to the similar demographics of the participants, it is possible that the new-idea plateau was reached sooner than if there we more diverse focus groups. It is probable that had students from the clinical phase also participated in the focus group, they would have been able to provide a more rounded view on how medicine may be in the year 2050, as they have had exposure to working in a hospital environment and are more knowledgeable about what being a doctor is like. Furthermore, by choosing mixed-sex focus group participants, it could have provided different data due to the possibility that the sexes may have different experiences in medical school so far. Another limitation is that the researcher belonged to the same cohort as the participants, which may have influenced the focus group discussions due to the dynamics associated with being perceived as a peer.

## Conclusions

The results revealed seven key areas in which medical students believed medical practice would change by 2050. They recognized that future practice will be shaped by both scientific advances and wider socioeconomic pressures. The future of the NHS was a major concern, as students felt that any changes to its structure or funding could significantly affect how medicine is organized and delivered. Students identified technology, AI, and genomics as the main scientific drivers of change, expressing cautious optimism about their potential to improve efficiency and patient outcomes while emphasizing that technology should support rather than replace doctors. They also acknowledged ongoing challenges such as antibiotic resistance, obesity, age-related illness, and the likelihood that new, unpredictable issues will emerge.

Students felt that the medical curriculum currently provides a solid foundation for future practice, with clinical experience viewed as its most valuable component. They agreed that medical education is a continuous process and that adaptability and lifelong learning will be essential as healthcare evolves. The study recommends that medical schools continue updating their curricula to include rapidly developing areas such as artificial intelligence, digital health, and genomics. Integrating these topics through case-based learning, simulation, and interprofessional activities would help students understand how to apply new technologies in both clinical and ethical contexts. Additional training in public health, preventive medicine, and healthcare systems would further prepare future doctors to manage challenges such as antibiotic resistance and chronic disease.

## Appendices

1.     What do you think some of the changes are in medicine that you might see in your career?

2.     Do you feel like by the time you graduate, you will have the knowledge to equip you to work as a doctor?

3.     What are some things that you wish you had learnt more of at medical school, or that you learnt that you have not done at all?

4.     Is there anything you would want to get rid of from the curriculum?

5.     What improvements do you think could be made in medical school, not just from a teaching perspective, to help you become better prepared to be a doctor?

6.     What are the biggest changes in medicine in the past 50 years that you are aware of? What are some technological advances in medicine that you think may occur by 2050? Do you think that any diseases or conditions will be more prevalent by 2050?

7.     Do you think that any diseases or conditions will be less prevalent by 2050?

8.     Do you feel that you are prepared for the changes that may occur in medicine by 2050? Have you ever heard of the Topol report?

9.     Have you learnt about these specific areas (genomics, AI, and technology) in medical school?

10.  Do you feel like it would help if you did?

11.  Would you like to learn about any of these things? Have you ever heard of Babylon?

12.  What are some of the pros and cons you can think of for services like Babylon? Do you think services like Babylon will increase in the future?

13.  Do you think wearable technology may change medicine within the next 30 years? Do you think that wearable technology will be useful in medicine?

14.  What do you think the future of genomics is within medicine?

15.  What do you think the future of artificial intelligence is within medicine?

## References

[1] Van Dellen JR (2012). Barber surgeon, or surgeon barber?. World Neurosurg.

[2] Clark G (1965). History of the Royal College of Physicians of London. BMJ.

[3] Van Way CW II (2022). War, medicine & death. Mo Med.

[4] Jefferson L, Bloor K, Maynard A (2015). Women in medicine: historical perspectives and recent trends. Br Med Bull.

[5] Grosios K, Gahan PB, Burbidge J (2010). Overview of healthcare in the UK. EPMA J.

[6] Logan AC, Prescott SL, Katz DL (2019). Golden age of medicine 2.0: lifestyle medicine and planetary health prioritized. J Lifestyle Med.

[7] Khan Z (2023). The emerging challenges and strengths of the National Health Services: a physician perspective. Cureus.

[8] Koehlmoos TP, Anwar S, Cravioto A (2011). Global health: chronic diseases and other emergent issues in global health. Infect Dis Clin North Am.

[9] Kulkova J, Kulkov I, Rohrbeck R, Lu S, Khwaja A, Karjaluoto H, Mero J (2023). Medicine of the future: how and who is going to treat us?. Futures.

[10] Conrad K (2023). The era of climate change medicine-challenges to health care systems. Ochsner J.

[11] van der Walt JL (2020). Interpretivism-constructivism as a research method in the humanities and social sciences - more to it than meets the eye. Int J Philos Theol.

[12] Krueger RA, Casey MA (2014). Focus groups: a practical guide for applied research. https://us.sagepub.com/en-us/nam/book/focus-groups-6.

[13] Topol E (2019). The Topol review. The Topol Review.

[14] Guest G, MacQueen KM, Namey EE (2014). Introduction to applied thematic analysis. Applied Thematic Analysis.

[15] Braun V, Clarke V (2021). Thematic Analysis: A Practical Guide. https://us.sagepub.com/en-us/nam/thematic-analysis/book248481.

[16] Holmes J (2021). Tackling Obesity: The Role of the NHS in a Whole-System Approach. https://www.kingsfund.org.uk/insight-and-analysis/reports/tackling-obesity-nhs.

[17] House of Lords (2017). The Long-Term Sustainability of the NHS and Adult Social Care. Authority of the House of Lords.

[18] Spence BS, Lambrechts MJ, Si Z, Leary EV, Gupta SK (2024). Telehealth visits have lower non-attendance than traditional orthopaedic clinic visits. Mo Med.

[19] Lakhani P, Sundaram B (2017). Deep learning at chest radiography: automated classification of pulmonary tuberculosis by using convolutional neural networks. Radiology.

[20] Mathur S, Sutton J (2017). Personalized medicine could transform healthcare. Biomed Rep.

[21] Zwart HA (2018). In the beginning was the genome: genomics and the bi-textuality of human existence. New Bioeth.

[22] (2019). No time to wait: securing the future from drug-resistant infections. https://www.who.int/publications/i/item/no-time-to-wait-securing-the-future-from-drug-resistant-infections.

[23] Jaul E, Barron J (2017). Age-related diseases and clinical and public health implications for the 85 years old and over population. Front Public Health.

[24] The Kings Trust (2018). Staffing shortfall of almost 250,000 by 2030 is major risk to NHS Long Term Plan, experts warn. Nuffield Trust.

[25] Ahmed SK, Mohammed RA (2025). Obesity: prevalence, causes, consequences, management, preventive strategies and future research directions. Metabol Open.

[26] Hruby A, Hu FB (2015). The epidemiology of obesity: a big picture. Pharmacoeconomics.

